# Routine MRI Signal Intensity as a Surrogate of Subchondral Bone Healing: Validation Against Micro-CT and Histology in an Ovine Model

**DOI:** 10.3390/diagnostics16152426

**Published:** 2026-07-31

**Authors:** Felix R. M. Koenig, Veronika Janacova, Markus Schreiner, Marlene Stuempflen, Vladimir Juras, Pavol Szomolanyi, Raoul Varga, Gregor Wollner, Janina M. Patsch, Giuseppe Filardo, Ali Guermazi, Siegfried Trattnig

**Affiliations:** 1 High-Field MR Center, Department of Biomedical Imaging and Image Guided Therapy, Medical University of Vienna, 1090 Vienna, Austria; 2 Department of Biomedical Imaging and Image Guided Therapy, Medical University of Vienna, 1090 Vienna, Austria; 3 Department of Orthopedics and Trauma-Surgery, Medical University of Vienna, 1090 Vienna, Austria; 4 Institute of Measurement Science, Slovak Academy of Sciences, 84104 Bratislava, Slovakia; 5 Faculty of Biomedical Sciences, Università della Svizzera Italiana, 6900 Lugano, Switzerland; 6 Chobanian & Avedisian School of Medicine, Boston University, Boston, MA 02118, USA; 7 Department of Radiology, VA Boston Healthcare System, West Roxbury, MA 02132, USA; 8 Austrian Cluster for Tissue Regeneration, 1040 Vienna, Austria; 9 Institute for Clinical Molecular MRI in the Musculoskeletal System, Karl Landsteiner Institute for Clinical Molecular MR, 1090 Vienna, Austria

**Keywords:** articular cartilage, basic cartilage and bone imaging, basic science in vivo animal research, scaffolds, histology, micro-CT, magnetic resonance imaging

## Abstract

**Background/Objectives:** To test whether routine T1-weighted spin-echo (T1-SE) and proton-density fast spin-echo (PD-FSE) MRI signal intensity (SI) can index bone regeneration after osteochondral scaffold implantation by correlating MRI metrics with micro-CT and histology. **Methods:** Twenty-eight sheep with bilateral trochlear defects were evaluated in separate cohorts at 30, 180, and 365 days (*n* = 7, 11, and 10, respectively). One knee received a tri-layered resorbable scaffold; the contralateral defect was left empty. MRI (T1-SE, PD-FSE) was read by two blinded musculoskeletal radiologists using 10-point Likert scales (T1: apparent mineralization; PD: SI normalization). Contrast-to-noise ratio (CNR) between repair-bone and reference bone was computed. MRI measures were correlated with micro-CT (new bone volume; trabecular bone volume) and histology (ICRS subchondral bone reconstruction; new bone; filling). **Results:** Likert ratings on both T1-SE and PD-FSE correlated with micro-CT new bone volume and trabecular bone volume and with histological measures of repair. PD-FSE CNR was inversely associated with micro-CT new bone volume and trabecular bone volume (both *p* < 0.001), indicating lower CNR with greater bone regeneration, whereas T1-SE CNR showed no significant associations. Inter-reader agreement was excellent (ICC 0.947 individual; 0.973 average), with good-to-excellent intra-reader reliability (0.920–0.963). **Conclusions:** Reader-based Likert assessment on both sequences and PD-FSE CNR provide complementary, non-invasive markers of subchondral bone regeneration, supporting MRI for radiation-free follow-up and endpoint selection in future translational studies. T1-SE CNR did not track incremental mineralization and should not be used as a stand-alone quantitative marker in early healing.

## 1. Introduction

The assessment of bone quality plays a critical role in understanding the progression of bone diseases and healing, especially in the context of osteochondral repair [1]. Advances in imaging technologies, including magnetic resonance imaging (MRI), have enabled more precise evaluations of bone quality and defect healing, reducing reliance on invasive techniques [2,3]. Continued developments in MRI hardware and acquisition have further improved bone contrast, positioning MRI as a radiation-free alternative to computed tomography for selected musculoskeletal indications [4,5]. MRI is a powerful, non-invasive tool that provides detailed insights into bone–cartilage interface as well as bone marrow, architecture and subchondral bone.

The use of T1-weighted sequences in MRI allows for detailed visualization of bone marrow and subchondral structures as signal intensity (SI) correlates negatively with bone mineral density (BMD). This property has been utilized to detect changes in bone quality, as in osteoporosis, where increased bone marrow adiposity is associated with lower BMD [6,7]. Historically, MRI has not been employed as the primary method for evaluating bone density due to the lack of standardized quantitative measures. However, recent studies demonstrated that MRI-based scoring systems, such as the vertebral bone quality score derived from T1-weighted images, can offer a reliable alternative to traditional bone density assessments like dual-energy X-ray absorptiometry (DXA) [8]. This scoring method has been validated in both clinical and experimental settings, particularly in osteoporotic and osteopenic conditions, correlating well with DXA and providing insights into bone quality changes [8,9].

Osteochondral implantation has become increasingly popular as a surgical option for addressing defects of the articular surface, particularly in younger patients [10]. MRI, which offers both structural and biochemical insights, is the standard modality for assessing cartilage quality after repair. There are several scores for follow-up MRI after cartilage repair, with the Magnetic Resonance Observation of Cartilage Repair Tissue (MOCART) Score 2.0 being the most commonly used scoring metric [11]. However, while MRI research has focused on the chondral component after cartilage repair, there is a gap regarding the bone component after osteochondral treatment. To date, it remains unclear how MRI performs in assessing bone quality in the context of osteochondral implantation; to our knowledge, no prior studies have directly validated routine MRI sequences against micro-CT or histology for bone regeneration. Specifically, the question arises as to whether MRI SI can effectively reflect the mineralization and structural integrity of bone following osteochondral implantation. This study aimed to establish whether MRI could be a valuable tool for assessing bone quality, by investigating its correlation with micro-CT and histological findings. This could potentially lead to the development of more refined, non-invasive diagnostic methods for bone health, especially in the context of orthopedic implants and bone regeneration treatments.

## 2. Methods

For this retrospective study, Fin-Ceramica (Faenza, Italy) provided micro-CT and histological data for the evaluation of bone quality in an ovine model, specifically focusing on the assessment of osteochondral repair in trochlear defects. The original study involved a 12-month Good Laboratory Practice safety and effectiveness evaluation of a porous, three-dimensional biomimetic, chemically and morphologically graded hybrid scaffold in an ovine model, conducted according to ASTM F2451-05 and ISO 10993-6 [12]. Animal care and surgery were reviewed and approved by the Testing Facility’s Institutional Animal Care and Use Committee (IACUC), in compliance with Canadian Council on Animal Care regulations, and performed at Charles River Laboratories Montreal ULC (Montreal, QC, Canada). The study was designed to assess the biocompatibility, safety, and efficacy of this osteochondral device for its potential clinical use in humans. The data from this study were provided for research purposes to evaluate bone quality using MRI techniques. The data from 30-, 180-, and 365-day time points were analyzed for changes in bone quality over time following the implantation of the device. This tri-layered biomimetic resorbable scaffold was composed of three distinct layers: a lower mineralized bone layer, an intermediate layer with reduced mineralization to replicate the tidemark, and an upper cartilage layer made from equine type I collagen [12]. More information about the study design and inclusion criteria is provided in the Supplementary Material.

**Figure 1:**
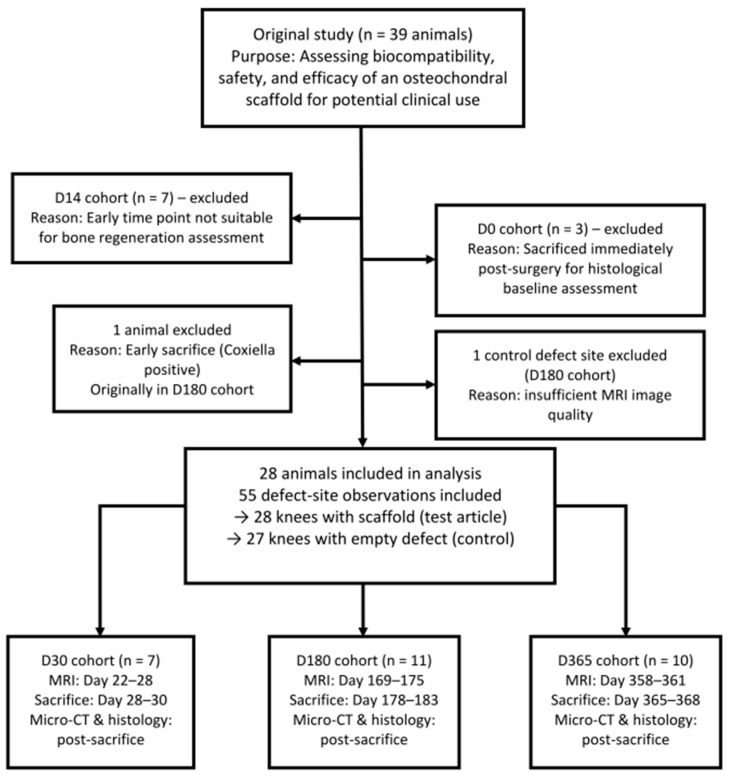
Study flow depicting inclusion and exclusion criteria.

Bilateral osteochondral defects were created in the trochlear groove of each of the 28 animals included in this study. One knee received the test article (Group 1: osteochondral scaffold), and the other knee served as control (Group 2: empty defect). Details are shown in Figure 1. More information is provided in the Supplementary Material. One control knee from animal D180-03 was excluded due to insufficient image quality. Because micro-CT and histology are destructive, each animal was evaluated at a single time point; the cohorts therefore comprised separate animals (30 days, *n* = 7; 180 days, *n* = 11; 365 days, *n* = 10), yielding 55 evaluable defect-site observations after the pre-specified exclusions.

### 2.1. MRI Methods

MRI of the operated hindlimbs from animals in the D30, D180, and D365 cohorts was performed using the Magnetom Sonata Syngo 1.5 Tesla system by Siemens (Erlangen, Germany). The images were acquired using Syngo MR XA30A software. The parameters of the MRI protocol and information about the MRI dataset are reported in the Supplementary Material. Sequences were originally optimized for morphological follow-up (including MOCART-related assessment); the semiquantitative Likert ratings and SI/contrast-to-noise ratio (CNR) analyses were subsequently leveraged to test their value as bone quality surrogates against micro-CT and histology.

### 2.2. Likert Scale

The MRI scans were evaluated in a blinded manner by a musculoskeletal radiologist (ST) with 35 years of experience. The following two Likert questions (1–10) were included to assess bone tissue repair.

1. Is the bone repair tissue signal intensity normal?—Likert: PD-FSE Signal Intensity
2. Is the bone repair tissue mineralized?—Likert: T1-SE Bone Mineralization

These Likert scores were independently verified by a second reader, a musculoskeletal radiologist resident (FRMK) with 4 years of experience. In cases of disagreement greater than 2 points between the two readers, a consensus was reached through joint review. No third adjudicator was used. For all other cases with minor discrepancies (≤2 points), the score of the senior musculoskeletal radiologist was used for analysis. The consensus or final senior score was then used for all correlation and modeling analyses. To assess intra-reader reliability, each reader re-evaluated a randomly selected subset of 10 MRI scans after a 6-week interval. Each 1–10 scale was anchored at both ends (1 = no evidence of the assessed feature; 10 = appearance indistinguishable from adjacent native subchondral bone). For “PD-FSE Signal Intensity,” readers graded the degree to which repair-tissue signal on fat-suppressed PD images had normalized toward the low, homogeneous signal of mature marrow-containing bone (higher scores denoting less residual edema-like/heterogeneous signal). For “T1-SE Bone Mineralization,” readers graded the extent of low-to-intermediate T1 signals and trabecular-like architecture indicating mineralized bone filling the defect (higher scores denoting more complete mineralization). Inter-reader concordance was high (inter-item correlation 0.949; intraclass correlation coefficient [ICC] 0.947 for individual and 0.973 for average measures), and intra-reader reliability was good to excellent (Reader 1 ICC 0.963; Reader 2 ICC 0.920); full values are reported in the Electronic Supplementary Material.

### 2.3. Contrast-to-Noise Measurements

In this study, CNR measurements were performed to quantitatively assess signal differentiation between newly formed bone tissue and surrounding structures. The analysis focused on three distinct regions of interest (ROIs):

ROI1: Signal intensity of the newly formed bone within the trochlea femoris.

ROI2: Signal intensity of the surrounding normal (native) subchondral/trabecular bone of the same trochlea, placed at least 1 cm from the defect margin in an unaffected region on the same slice, serving as the internal reference bone.

σ: Surrounding air, used as a background reference for noise estimation.

These measurements were performed on an axial PD-w FSE fat-suppressed (fs) and on a sagittal T1-w FSE without fs.

CNR was calculated by the following formula: CNR = ∣S(ROI1) − S(ROI2)∣/σ.

### 2.4. Micro-CT Analysis

Micro-CT imaging was performed on the defect sites of all included animals. For all samples, imaging was conducted using a SkyScan 1272 scanner (Bruker, Kontich, Belgium) with a resolution of 10.8 µm. The samples were scanned at room temperature and subsequently returned to 10% neutral-buffered formalin for preservation. More information is provided in the Supplementary Material.

The following data were captured for each implanted site:

- Trabecular bone volume (BV/TV, %)
- Defect area (mm^3^)
- Amount of new bone (mm^3^)

### 2.5. Histological Analyses

Histological evaluations were independently performed by Charles River Laboratories Montreal ULC (Quebec, Canada). See the Supplementary Material for more information.

The following variables were analyzed:

ICRS II Parameter

- Subchondral bone reconstruction

For the evaluation of the femoral bone–cartilage defect sites, the International Cartilage Repair Society (ICRS) II histological scoring parameter was used, with a focus on subchondral bone abnormalities.

Additional histological information

- New bone formation

This parameter quantifies the amount of newly formed bone tissue within the defect area, excluding the presence of bone marrow. It reflects the extent of mineralized tissue present.

- Filling with new bone/marrow

This parameter assesses the extent to which the defect is filled by newly formed bone and marrow, including marrow tissue located between the trabecular bone, if present.

### 2.6. Statistical Analysis

Statistical analysis was performed using R version 4.4.1 (R Foundation for Statistical Computing) in RStudio version 2024.04.2 (RStudio, PBC), and IBM SPSS version 24.0.1 for Windows (IBM, Chicago, IL, USA). More information is provided in the Supplementary Material. Because defect-site observations were clustered within animals and cohorts differed by follow-up time, non-independence was addressed using mixed-effects models with a random intercept for animal (cumulative link mixed models for ordinal Likert outcomes; linear mixed-effects models for CNR); Spearman correlations are reported with 95% confidence intervals. A concise summary of correlation-strength interpretation and the modeling framework is provided below and in full in the Electronic Supplementary Material.

## 3. Results

### 3.1. Data Distribution

**Figure 2:**
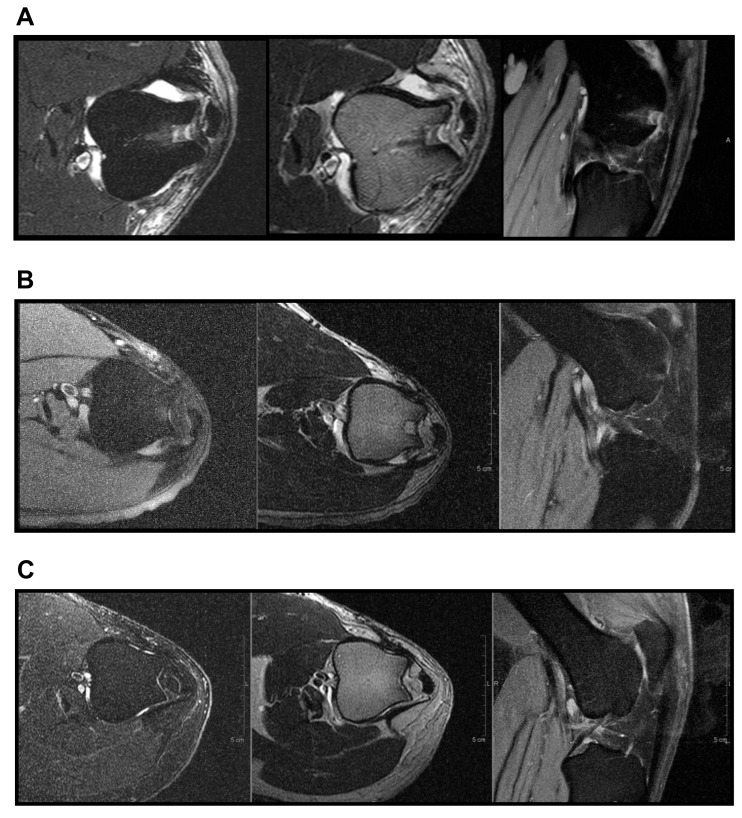
Reference MRI images, from left to right: axial PD fs, axial T1 without fs, sagittal PD fs. (**A**): MRI after 30 days, “PD Signal Intensity” was rated 3, “T1 bone mineralization” was rated 2. (**B**): MRI after 180 days, “PD Signal Intensity” was rated 7, “T1 Bone Mineralization” was rated 6. (**C**): MRI after 365 days, “PD Signal Intensity” was rated 10, “T1 Bone Mineralization” was rated 10.

The data from the study were analyzed for two primary Likert questions concerning bone repair tissue: (1) “Is the bone repair tissue signal intensity normal?—PD Signal Intensity Likert”, and (2) “Is the bone repair tissue mineralized?—T1 Bone Mineralization”. Summary statistics for Likert questions, micro-CT and histological variables across time points are shown in the Supplementary Material (30 days, *n* = 7; 180 days, *n* = 11; 365 days, *n* = 10; 55 evaluable defect-site observations). The interrater reliability analysis showed excellent agreement between the two readers regarding the Likert rating (Supplementary Material). Figure 2 shows reference MRI after 30, 180, and 365 days.

### 3.2. Correlations

The correlation analysis between MRI ratings and micro-CT and histological parameters revealed significant positive correlations (Table 1). Specifically, “PD Signal Intensity” showed a correlation coefficient of 0.55 with “Amount of new bone” (*p* < 0.001) and 0.54 with “Trabecular bone volume” (*p* < 0.001). Additionally, a strong positive correlation was observed between “PD Signal Intensity” and histology of “Subchondral bone reconstruction” (0.60, *p* < 0.001). The correlation between “PD Signal Intensity” and “New bone formation” was not significant. With “PD Signal Intensity” and “Filling with new bone/marrow”, the correlation coefficient was 0.46 (*p* < 0.001). Correlation strength was interpreted as very weak (0.00–0.19), weak (0.20–0.39), moderate (0.40–0.59), strong (0.60–0.79), and very strong (≥0.80).

For the T1 bone mineralization Likert of bone repair tissue, the analysis showed similarly strong correlations (Table 2). Mineralization was positively correlated with “Amount of new bone” (0.70, *p* < 0.001) and “Trabecular bone volume” (0.70, *p* < 0.001). “Subchondral bone reconstruction” had a strong correlation with “T1 Bone Mineralization” (0.68, *p* < 0.001), while “New bone formation” (histology) and “Filling with new bone/marrow” showed correlation coefficients of 0.29 (*p* = 0.033) and 0.55 (*p* < 0.001), respectively.

**Figure 3:**
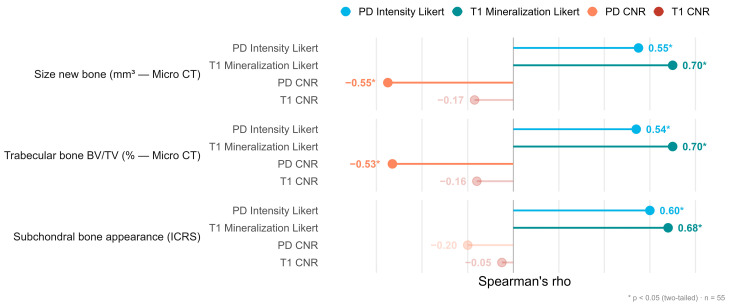
Overview of Spearman correlations (ρ) between each MRI metric (PD Intensity Likert, T1 Mineralization Likert, PD CNR, T1 CNR) and the reference standards: micro-CT new bone volume, micro-CT trabecular bone volume (BV/TV), and ICRS subchondral bone appearance. Positive values indicate agreement with greater bone regeneration; PD CNR is inversely related to micro-CT. Asterisks denote *p* < 0.05 (two-tailed); *n* = 55.

**Figure 4:**
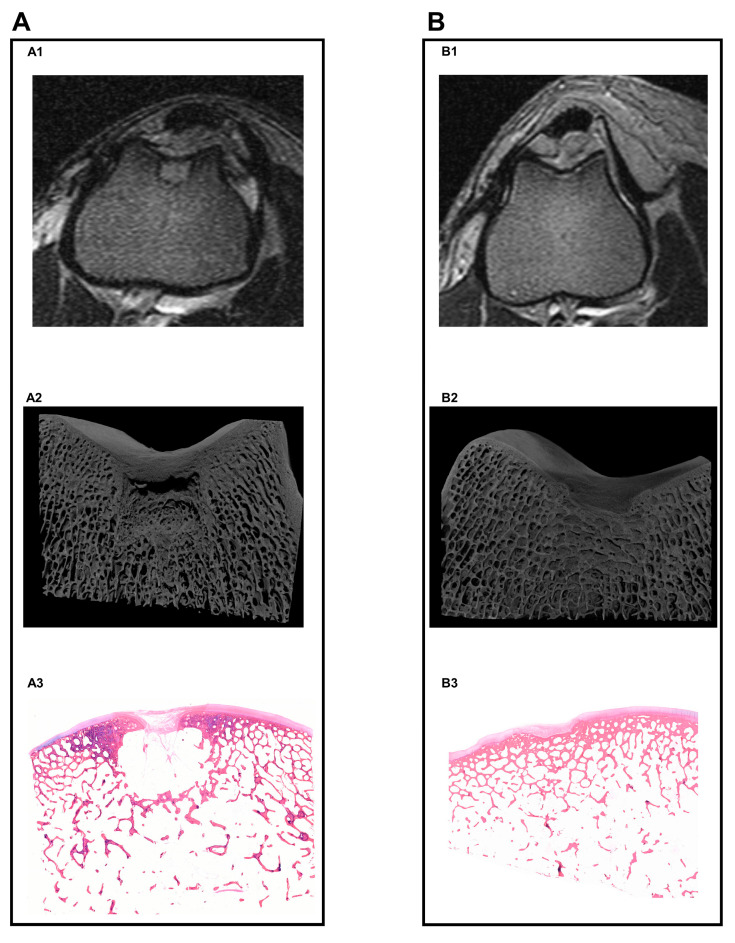
(**A**): 180 d, left femoral trochlea, top to bottom: T1 FSE axial—micro-CT 3D reconstruction coronal—H&E, not decalcified, original magnification 2.5×. (**A1**): This MRI was scored 6/10 for “T1 bone mineralization” Likert. (**A2**): Micro-CT: “Amount of new bone” was 66.85 mm^3^, “Trabecular bone volume” was 27.40%. (**A3**): “Subchondral bone reconstruction” was histologically scored 10%, “New bone formation” was 1 (minimal) on the 0–4 scale. (**B**): 365 d, left femoral trochlea, top to bottom: T1 FSE axial—micro-CT 3D reconstruction coronal—H&E, not decalcified, original magnification 0.4×. (**B1**): This MRI was scored 10/10 for “T1 Bone Mineralization” Likert. (**B2**): Micro-CT: “Amount of new bone” was 95.99 mm^3^, “Trabecular bone volume” was 40.10%. (**B3**): Histology: “Subchondral bone reconstruction” was scored 90%, “New bone formation” was 4 (marked) on the 0–4 scale.

Only CNR calculated from PD-FSE weighted images showed a correlation with micro-CT parameters. Specifically, it correlated with “Amount of new bone (mm^3^)” (−0.55, *p* < 0.001) and “Trabecular bone volume BV/TV (%)” (−0.53, *p* < 0.001). Details are shown in the Supplementary Material. Figure 3 provides an overview of the correlations of all MRI metrics with the reference standards. Figure 4 shows exemplary MRI-SI correlation with micro-CT and histology.

### 3.3. Cumulative Link Mixed Models (CLMMs) Likert—Micro-CT Summary

T1 bone mineralization was positively and significantly associated with trabecular bone volume. Notably, each 1 mm^3^ increase in new bone volume was associated with 4.5% higher odds of a higher T1 mineralization score. Each percentage-point increase in BV/TV was associated with 10.1% higher odds of a higher T1 score. The association between BV/TV and the T1 score was stronger in the scaffold group. Full statistical model outputs are provided in the Supplementary Material and in Table 3.

### 3.4. CLMM Likert—Histology Summary

**Figure 5:**
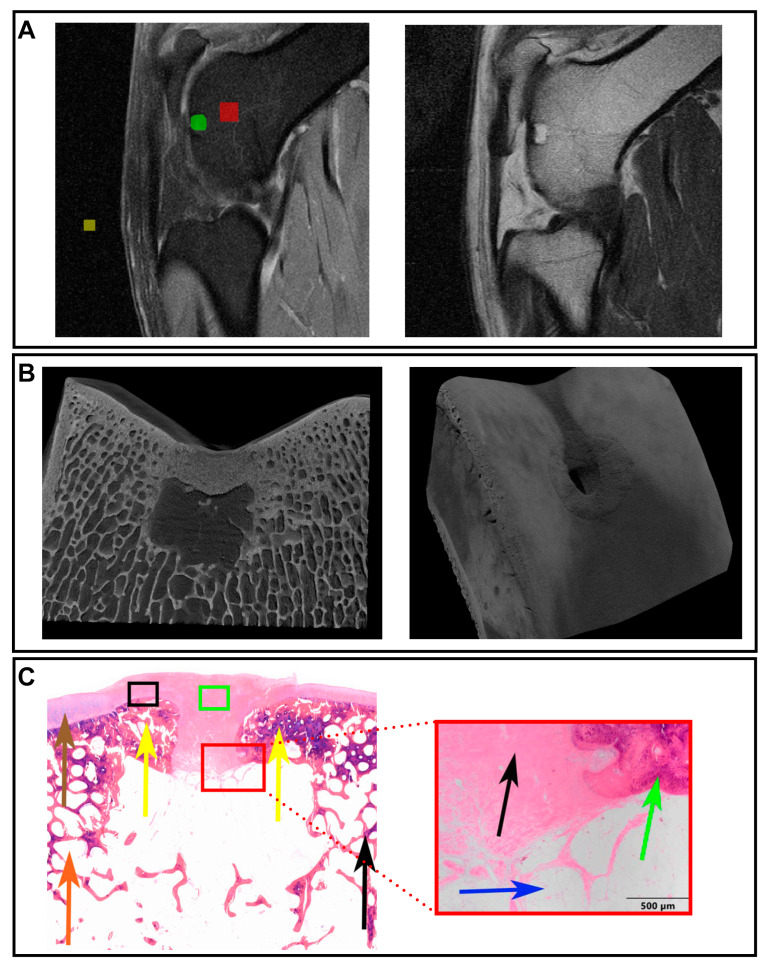
365 d, right femoral trochlea, left to right: (**A**): Left: PD fs sagittal with CNR measurements—Green: Bone defect with regeneration tissue. Red: Reference Bone, Yellow: Standard deviation of the background noise. PD-FSE CNR: 2.6; Right: T1 sagittal. The bone defect area appears clearly hyperintense in comparison to surrounding bone—Mineralization Likert 6/10. (**B**): Left: Micro-CT 3D reconstruction coronal; Right: Micro-CT 3D reconstruction; There is no full filling of the osseous defect centrally. The maximum ossification is at the upper end of the defect. Size new bone (mm^3^) = 82.07; (BV/TV) = 34.10. (**C**): Left: Non-decalcified section stained with H&E, original magnification 0.4×. The black arrow indicates pre-existing bone, while the brown arrow marks pre-existing cartilage. New bone formation is visible at the defect margins, located beneath the superficial defect layer (yellow arrows). The red box highlights an area selected for high magnification. In the central region of the defect, the superficial layer consists of fibrous tissue (green box). At the defect edges, the superficial zone comprises fibrocartilage-like tissue in the deeper portion (black box) and fibrous tissue at the surface. The orange arrow points to the surrounding bone marrow. Right: Right femoral trochlea, non-decalcified, H&E stain, magnification 4×. The green arrow marks newly formed bone, the black arrow indicates adjacent fibrous tissue, and the blue arrow denotes adipose tissue. “Subchondral bone reconstruction” = 25/100; “New bone formation (Histology)” = 1.5/5; “Filling with new bone/marrow” = 1.5/5.

Due to collinearity, the treatment group variable was excluded from the final models. Histological subchondral bone reconstruction significantly predicted higher PD signal intensity and T1 bone mineralization scores. Histological new bone formation significantly predicted higher T1 bone mineralization scores (OR = 5.176, 95% CI: 2.145–12.490, *p* < 0.001), whereas the PD signal intensity–new bone formation model was not fitted because the underlying bivariate relationship was not significant. Filling with new bone/marrow significantly predicted higher PD signal intensity scores (OR = 5.098, 95% CI: 2.666–9.748, *p* < 0.001); for T1 bone mineralization, the squared filling term was significant (OR = 1.373, 95% CI: 1.214–1.554, *p* < 0.001), reflecting the transformed predictor used to satisfy the proportional-odds assumption. Full model results and tables are provided in the Supplementary Material. To illustrate the full spectrum of healing, Figure 2 depicts the temporal progression from low to high scores (30, 180, and 365 days), and Figure 4 contrasts a lower-scoring case (A) with a well-regenerated, high-scoring case (B), validated against micro-CT and histology. Figure 5 shows the 365-day status of the right femoral trochlea, combining PD-FS and T1 MRI, micro-CT reconstruction, and histology.

### 3.5. Linear Mixed-Effects Models CNR PD Summary

For the outcome “Amount of new bone”, the fixed effects showed a significant negative relationship between the CNR PD calculation and new bone size (Estimate = −5.54, 95% CI: −7.91 to −3.16, *p* < 0.001), indicating that for every unit increase in CNR PD calculation, the amount of new bone decreases by 5.54 mm^3^.

For the outcome “Trabecular bone volume BV/TV (%)”, the results showed a significant negative effect of CNR PD calculation on trabecular bone volume fraction (Estimate = −2.35, 95% CI: −3.47 to −1.24, *p* < 0.001), indicating that for every unit increase in CNR PD calculation, the trabecular bone volume decreases by 2.35% (details in Table 4 and in the Supplementary Material).

## 4. Discussion

This study addresses a notable gap in musculoskeletal imaging: MRI-based evaluation of subchondral bone healing in osteochondral repair. Beyond morphology, we demonstrate that routine reader-based PD-FSE and T1-SE signal assessments track bone regeneration and are validated against micro-CT architecture and histopathology. Likert scores on both sequences correlated with reference-standard measures, whereas only PD-FSE CNR was inversely associated with micro-CT bone parameters; T1-SE CNR showed no significant associations. These findings support a practical, method-specific role for routine MRI in assessing the osseous component of osteochondral scaffold healing.

### 4.1. PD-FSE SI Abnormalities

Previous reports indicated that T2 SI on PD fs, STIR, and T2 fast spin-echo-weighted fs sequences are highly sensitive for detecting fluid, highlighting bone marrow edema-like signal as hyperintense against suppressed fat [13].

Our quantitative results indicate that higher PD CNR values are significantly and inversely correlated with bone quality parameters from micro-CT, including new bone volume and trabecular bone volume. Strong significant correlations were also observed between the histological ICRS subchondral bone abnormalities and PD SI Likert rating. Similarly, Chang et al. reported significant correlations between quantitative proton-density values generated by synthetic MRI and lumbar-spine DXA T-scores, further supporting the sensitivity of PD SI for detecting bone mineralization changes [14]. This suggests that the increased PD SI in regenerating bone tissue reflects a combination of factors, including bone remodeling, mineralization changes, and residual marrow edema. These observations are in line with prior studies showing that PD-weighted MRI can detect bone mineralization deficiencies in osteoporotic conditions [15], and support its use for non-invasive evaluation of subchondral bone regeneration. Our findings support the hypothesis that bone regeneration can be effectively evaluated through PD-weighted MRI SI when compared with the surrounding bone structure.

### 4.2. T1-SE SI Abnormalities

T1-weighted imaging is a valuable tool to assess bone quality and its mineralization status, as demonstrated in studies on bone fracture healing [16]. T1 relaxation time of bone is sensitive to its mineral content, and changes in T1 SI can reflect important biochemical and structural alterations in the bone tissue during repair. In the healing process of fractures, for example, the T1 values decrease over time as bone matures and mineralizes, providing a quantitative way to track these changes noninvasively [16].

Similar principles hold true for osteochondral implants. Studies indicate an association between bone healing and mineralization with significant changes in T1 values. Particularly, T1-weighted MRI has been proven to be a sensitive tool for evaluating bone mineralization, as demonstrated in studies of osteoporosis, reporting an inverse relationship between BMD and T1-weighted images [8].

We found significant correlations between bone mineralization ratings and histology as well as micro-CT: T1-weighted Likert scores showed stronger correlations with micro-CT bone parameters than PD-weighted scores, particularly for trabecular bone volume and new bone amount. This finding aligns with the work of Trattnig et al., who emphasized that high-resolution MRI and especially T1-weighted sequences could offer critical information regarding subchondral bone health after osteochondral procedures [17]. Moreover, as shown in the work by Ehresman et al., MRI-based techniques can significantly aid in diagnosing BMD changes, which are indicative of osteoporotic bone quality [8]. This is particularly relevant for osteochondral implantation, where both graft and subchondral bone quality are crucial to the long-term success of the procedure.

### 4.3. Semiquantitative Morphological Assessment and CNR

However, despite semiquantitative T1 SI Likert scores showing strong, significant correlations with micro-CT and histological parameters, the T1 CNR measurements did not reach statistical significance. This was unexpected, as T1-based CNR has been used elsewhere to quantify bone healing and mineralization (e.g., significant negative correlations with DXA M- and T-scores reported by Chang et al. in vertebral degeneration [14]). Unlike that setting, our model targets regenerating tissue in which newly formed bone undergoes a gradual transition of marrow composition; early remodeling with residual edema and mixed fibrous/fatty components can heterogenize T1 signal within the defect [16]. Methodologically, the Likert approach allows expert readers to integrate such contextual cues—spatial patterns of marrow signal, interface morphology, and comparison to adjacent bone—yielding a semiquantitative score sensitive to subtle, patchy remodeling. In contrast, an ROI-based CNR reduces this complexity to a single contrast value, which can average out small-scale variations and thus fail to distinguish partially mineralized tissue from surrounding marrow in early healing. This interpretation is supported by the significant T1 Likert correlations with both histological new bone formation and filling with new bone/marrow, indicating partially overlapping signal characteristics during scaffold maturation. Although T1 CNR was not significant, the consistent Likert correlations underscore that expert semiquantitative assessment captures biologically relevant changes that pure numeric CNR may miss, and more closely reflects clinical MRI reading. A further, sequence-specific contribution is that non-fat-suppressed T1-SE offers a comparatively narrow dynamic range between partially mineralized repair tissue and adjacent fatty marrow; the high native marrow signal can compress the measurable contrast (an effective ceiling/saturation effect), so a single ROI-based T1 CNR is less able to resolve incremental mineralization than the fat-suppressed PD-FSE contrast or an integrative reader score. Accordingly, T1-SE CNR should be considered exploratory and should not be relied upon as a stand-alone marker of incremental mineralization during early healing.

### 4.4. Bone Assessment in MRI

In clinical practice, CT is often used to assess bone quality, while cartilage regeneration is regularly followed up with MRI [17]. Our data indicate that standard PD-FSE and T1-SE weighted MRI can also capture biologically meaningful changes in the osseous component. Thus, within a single MRI session, clinicians can obtain complementary information on cartilage and subchondral bone without additional procedures or radiation, supporting MRI signal intensity as a structural surrogate for bone regeneration. These findings are consistent with other studies that demonstrated the utility of MRI in assessing bone quality and predicting outcomes in cartilage repair procedures [9]. Given that micro-CT is widely regarded as the gold standard for quantifying bone structure and volume, the correlations of PD-FSE and T1-SE Likert ratings, together with the inverse PD-FSE CNR associations, support MRI’s relevance as a structural imaging surrogate; however, T1-SE CNR alone was not informative in this setting [18,19]. Although advanced bone-imaging sequences (e.g., ultrashort echo time or zero echo time) were not used here, the performance of reader-based PD-FSE and T1-SE assessment, together with PD-FSE CNR, enhances clinical translatability, supporting future clinical translation into postoperative follow-up and research workflows.

### 4.5. Human vs. Ovine Knee: Translational Value and Model Limitations

The ovine osteochondral defect model offers meaningful translational value for human applications: sheep provide comparable joint dimensions that accommodate human-sized implants and enable controlled testing of osteochondral repair strategies in a large-animal setting, as emphasized in comprehensive reviews of osteochondral models [20]. Anatomically, the ovine stifle closely mirrors the human knee (effectively a ~1/3 scale for many bony measures), supporting its use for preclinical imaging–biomarker validation; nonetheless, relevant differences include a narrower femoral intercondylar notch, slimmer trochlear groove, and thicker proximal tibial cortex, each with implications for biomechanics and healing [21].

These species differences frame both the strength and the boundary of translation. Unlike human postoperative protocols with restricted weight-bearing, sheep resume full loading immediately, and ovine trochlear lesions often heal more efficiently than analogous human defects—factors that can accelerate remodeling and confound direct extrapolation of temporal healing trajectories [20]. Accordingly, our data should be viewed as preclinical proof-of-concept that MRI signal intensity can index subchondral bone healing, motivating hypothesis-driven human studies rather than implying immediate clinical equivalence. Long-term clinical outcomes (e.g., implant longevity, osteoarthritis prevention) remain uncertain without prospective human validation, yet these results—anchored by correlations with micro-CT and histology—provide a solid platform for designing such trials.

Our study has limitations. Semiquantitative Likert grading may introduce reader-dependent variability, although we mitigated this with two blinded, independent readers and demonstrated strong inter-/intra-reader agreement. Our follow-up ended at 1 year, so we cannot determine whether marrow-rich defects subsequently fully mineralize, limiting long-term inference. Finally, ovine bone mineral density and remodeling kinetics differ from those of humans: sheep remodel bone more rapidly and resume full weight-bearing immediately after surgery, whereas human protocols typically restrict loading. Consequently, the temporal trajectory of PD-FSE signal normalization and T1 mineralization observed here is likely compressed relative to human clinical timelines, and the specific time points should not be transferred directly to patients; the validated signal–structure relationships, rather than their absolute timing, are the translatable finding and should be confirmed against human follow-up intervals in prospective studies.

In conclusion, our study supports routine MRI as a non-invasive, radiation-free approach for assessing bone quality after osteochondral implantation. Reader-based Likert evaluation on both PD-FSE and T1-SE provided complementary information on mineralization and reparative tissue signal, while lower PD-FSE CNR was associated with greater new bone volume and BV/TV. In contrast, T1-SE CNR showed no significant associations with micro-CT or histology and should not be relied upon as a stand-alone quantitative marker of incremental mineralization during early healing. Prospective human studies are needed to validate these sequence- and method-specific MRI markers and determine their value for postoperative management.

## 5. Key Results

- Standardized Likert-based signal intensity ratings on both PD-FSE and T1-SE correlated with micro-CT parameters and selected histological bone quality parameters, supporting their value for assessing bone regeneration.
- PD-FSE CNR was inversely associated with micro-CT new bone volume and BV/TV, indicating lower CNR with greater bone regeneration; however, T1-SE CNR did not reliably reflect incremental mineralization.
- The validation of MRI signal intensity against histological and micro-CT outcomes supports MRI as a clinically relevant, non-invasive tool for monitoring bone regeneration in osteochondral scaffold procedures.

### Summary Statement

MRI signal intensity—assessed visually on PD-FSE and T1-SE and supplemented by PD-FSE CNR—correlates with subchondral bone regeneration after osteochondral scaffold implantation, supporting MRI as a non-invasive tool without ionizing radiation for assessing mineralization and structural bone integrity; however, T1-SE CNR alone was not a reliable quantitative marker of early mineralization.

## Figures and Tables

**Table 1:** Correlation of PD-FSE weighted MRI (Likert) with Micro-CT and Histology.

PD Signal Intensity	ρ (95% CI)	Correlation Strength	Significance (2-Tailed)	*n*
Amount of new bone (mm^3^)—Micro-CT	0.55 (0.33–0.71)	Moderate	* < 0.001	55
Trabecular bone volume BV/TV (%)—Micro-CT	0.54 (0.32–0.70)	Moderate	* < 0.001	55
Subchondral bone reconstruction—ICRS Histology	0.60 (0.40–0.75)	Strong	* < 0.001	55
New bone formation—Histology	0.17 (–0.10–0.42)	Very Weak	0.221	55
Filling with new bone/marrow—Histology	0.46 (0.22–0.65)	Moderate	* < 0.001	55

* *p* < 0.05 (two-tailed).

**Table 2:** Correlation of T1-SE weighted MRI (Likert, “Bone Mineralization”) with Micro-CT and Histology.

T1 Bone Mineralization	ρ (95% CI)	Correlation Strength	Significance (2-Tailed)	*n*
Amount of new bone (mm^3^)—Micro-CT	0.70 (0.53–0.81)	Strong	* < 0.001	55
Trabecular bone volume (BV/TV, %)—Micro-CT	0.70 (0.53–0.81)	Strong	* < 0.001	55
Subchondral bone reconstruction—ICRS Histology	0.68 (0.51–0.80)	Strong	* < 0.001	55
New bone formation—Histology	0.29 (0.03–0.52)	Weak	* 0.033	55
Filling with new bone/marrow—Histology	0.55 (0.33–0.71)	Moderate	* < 0.001	55

* *p* < 0.05 (two-tailed).

**Table 3:** Random and fixed effects of cumulative link mixed models describing the relationship of the Likert score and micro-CT parameters controlled for the intervention group.

PD Signal Intensity					T1 Bone Mineralization				
Amount of new bone (mm^3^)					Amount of new bone (mm^3^)				
*Random effects*					*Random effects*				
	Variance	SD				Variance	SD		
Animal	2.616	1.617			Animal	0.775	0.880		
*Fixed effects*					*Fixed effects*				
	OR	Lower CI	Upper CI	*p*-value		OR	Lower CI	Upper CI	*p*-value
Amount of new bone	1.026	1.000	1.053	0.055	Amount of new bone	1.045	1.022	1.069	<0.001
Group (1)	0.070	0.002	2.254	0.133	Group (1)	0.049	0.002	1.354	0.075
Amount of new bone: Group (1)	1.057	1.016	1.099	0.006	Amount of new bone: Group (1)	1.050	1.011	1.090	0.012
Trabecular bone volume (BV/TV, %)					Trabecular bone volume (BV/TV, %)				
*Random effects*					*Random effects*				
	Variance	SD				Variance	SD		
Animal	2.615	1.617			Animal	0.932	0.965		
*Fixed effects*					*Fixed effects*				
	OR	Lower CI	Upper CI	*p*-value		OR	Lower CI	Upper CI	*p*-value
Trabecular bone volume	1.056	0.999	1.118	0.056	Trabecular bone volume	1.101	1.047	1.158	<0.001
Group (1)	0.118	0.004	3.202	0.204	Group (1)	0.082	0.003	2.007	0.125
Trabecular bone volume: Group (1)	1.122	1.031	1.221	0.008	Trabecular bone volume: Group (1)	1.106	1.019	1.200	0.016

**Table 4:** Linear mixed-effects models of CNR PD and micro-CT variables.

Amount of New Bone (mm^3^)—Micro CT					Trabecular Bone Volume BV/TV (%)—Micro CT				
*Predictors*	*Estimates*	*CI*	*p*	*df*	*Predictors*	*Estimates*	*CI*	*p*	*df*
(Intercept)	95.55	81.37–109.73	<0.001	46.19	(Intercept)	42.04	35.34–48.73	<0.001	46.33
CNR PD Calculation	−5.54	−7.91–−3.16	<0.001	51.99	CNR PD Calculation	−2.35	−3.47–−1.24	<0.001	51.88
Group	17.14	7.76–26.52	0.001	36.19	Group	7.14	2.78–11.49	0.002	36.02
Random Effects					Random Effects				
σ2	186.44				σ2	39.71			
τ00 AnimalID				485.5	τ00 AnimalID				112.03
ICC	0.72				ICC	0.74			
N AnimalID	28				N AnimalID	28			
Observations			55		Observations			55	
Marginal R2/Conditional R2			0.433/0.843		Marginal R2/Conditional R2			0.377/0.837	

## Data Availability

The datasets analyzed during the current study are not publicly available because the underlying micro-CT and histological data are the property of a third party (Fin-Ceramica Faenza S.p.A., Faenza, Italy) and were provided to the authors for research purposes. Data are available from the corresponding author(s) on reasonable request and with the permission of the third party.

## Supplementary Materials

The following supporting information can be downloaded at: https://www.mdpi.com/article/10.3390/diagnostics16152426/s1, extended Methods (animal model and study design, MRI protocol parameters [Table S1], micro-CT and histological analyses, statistical analysis), extended Results, and supplementary tables. Table S1: Parameters of the MRI Protocol; Table S2: Summary statistics for Likert questions, micro-CT and histological variables across time points (D30), (D180), and (D365); Table S3: Correlation of PD-based Contrast-to-Noise Ratio (CNR) with Micro-CT and Histology; Table S4: Correlation of T1-based Contrast-to-Noise Ratio (CNR) with Micro-CT and Histology; Table S5: Random and fixed effects of cumulative link mixed models describing the relationship of the Likert score and Histology parameters. References [12,22,23,24,25] are cited in the Supplementary Materials.

## Abbreviation and Acronyms

BMD	Bone mineral density
CLMMs	Cumulative link mixed models
CNR	Contrast-to-noise ratio
DXA	Dual-energy X-ray absorptiometry
fs	Fat-suppressed
MRI	Magnetic resonance imaging
MOCART	Magnetic Resonance Observation of Cartilage Repair Tissue
PD-FSE	Proton-density fast spin-echo
SI	Signal intensity
T1-SE	T1-weighted spin-echo
