# Supplementary material for: Routine MRI Signal Intensity as a Surrogate of Subchondral Bone Healing: Validation Against Micro-CT and Histology in an Ovine Model
Source: Diagnostics (Basel). 2026 Jul 31;16(15):2426. doi: 10.3390/diagnostics16152426 (PMC13464844; doi:10.3390/diagnostics16152426)
Supplement: Supplementary file 1 [file diagnostics-16-02426-s001.zip › diagnostics-4415477-supplementary.pdf]

Electronic Supplementary Material

Routine MRI Signal Intensity as a Surrogate of Subchondral Bone Healing: Validation against Micro-CT and Histology in an Ovine Model

Table of Content

Methods (Extended) 1
Results (Extended): 9
List of Tables - Electronic Supplementary Material 15

Methods (Extended)

Animal Model and Study Design

The original animal study was performed at Charles River Laboratories Montreal (Quebec, Canada) under Good Laboratory Practice (GLP) conditions, in compliance with U.S. FDA regulations (21 CFR Part 58) and OECD Principles of GLP. Experimental design and conduct adhered to ASTM F2451-05 for evaluation of osteochondral repair devices and aligned with ICRS guidance [22,23]. Sheep (Ovis aries) were selected as a functional in-vivo anatomical model recommended by ASTM F2451-05 and ICRS guidelines, given their joint dimensions and biomechanics suitable for testing human-sized osteochondral implants. MRI was included as part of the comprehensive assessment of bone quality and defect healing in collaboration with our center; all image rating, quantitative analyses, and correlative statistics were performed independently at our institution.

A total of 39 animals were included in the original study. For this retrospective analysis, however, only 28 animals were considered: 10 animals, that were sacrificed at 0 and 14 days in the original study, were excluded from this analysis. Specifically, three animals were sacrificed immediately after surgery (Day 0) and used for histology baseline assessments. In these animals, a defect was created in the trochlear groove of one limb and filled with the test article. The defect site was returned to Charles River for biomechanical testing and histopathological evaluation of baseline biodegradation. Seven animals were sacrificed at 14 days postoperatively and were excluded from the analysis, as this early time point was not suitable for evaluating bone regeneration. Additionally, one animal (which had tested positive for Coxiella bacteria) from the 180-day group was excluded due to an early sacrifice. One control knee from animal D180-03 was excluded due to insufficient image quality. No necropsy was performed following the veterinarian's instructions and the study director's approval, and the carcass was disposed of accordingly. Surgical, anaesthetic and euthanasia procedures were performed by the Testing Facility in the primary study [12]. Anaesthesia for surgery was induced with midazolam and ketamine with glycopyrrolate premedication and maintained with inhalational isoflurane in oxygen under

mechanical/manual ventilation after intubation; perioperative antibiotic prophylaxis (cefazolin) was administered. Through a medial parapatellar arthrotomy, critical-sized cylindrical osteochondral defects (approximately 7 mm diameter and 5.5 mm depth) were created in each trochlear groove using a customized instrumentation kit; the scaffold was press-fitted bone-side down in one knee, while the contralateral defect was left empty as control, and four micro-perforations were made at the defect base to enhance bleeding. Postoperative analgesia/antibiosis included duplocillin and carprofen (days 2 and 4); surgical sites were assessed for lameness, body condition and pain daily during the first postoperative week and weekly thereafter until wound healing and normal behaviour were observed. Animals were group-housed under controlled temperature, humidity and light cycle with access to hay, food and water, with smaller enclosures postoperatively to limit brisk activity. At the scheduled endpoint animals were euthanised by intravenous sodium pentobarbital, after which the stifle joints were harvested for micro-CT and histological analysis.

MRI Methods

To adequately capture the implanted sites, the following imaging sequences were used. To minimize animal distress and avoid excessive movements, all animals were sedated prior to the MRI examination: For the imaging session the operated hind limbs were positioned and immobilised within the coil to reduce motion, and datasets were reviewed for diagnostic quality; scans degraded by motion were not used for scoring. Because MRI was acquired ex-cohort prior to the scheduled sacrifice at each time point, no longitudinal re-imaging of the same animal was performed (see cohort disposition below).

- T2-weighted fast spin-echo with fat-suppression in the axial plane
- T2-weighted fast spin-echo without fat-suppression in the axial plane
- Proton density-weighted fast spin-echo with fat-suppression in the sagittal plane
- Proton density-weighted fast spin-echo with fat-suppression in the axial plane
- T1-weighted fast spin-echo in the sagittal plane
- T1-weighted fast low angle shot (FLASH)-3D in the sagittal plane

The MRI scans were processed and analyzed to generate images that helped assess these parameters, contributing to the understanding of how the device supports osteochondral healing. The final dataset, including 2D and 3D images of the bone defects at various time points, was used to conduct this retrospective analysis. In this study, only data from the following MRI sequences were analyzed (Table S1).

Table S1. Parameters of the MRI Protocol

Sequence	PD-w TSE FS	T1-w TSE	T2-w TSE
Orientation	Axial	Sagittal	Axial
Voxel Size (mm)	0.3x0.3x3.0	0.4x0.4x3.0	0.6x0.5x2.2
Slice Thickness (mm)	3.0	3.0	2.2
TR (ms)	3000	649	4280
TE (ms)	16	16	66
Averages	2	1	2
FoV Read (mm)	120	135	120
FoV Phase (%)	100	100	100
Phase Oversampling (%)	100	100	100
Flip Angle (deg)	150	150	150
Fat Suppression	Yes	None	None
Bandwidth (Hz/Px)	126	114	65
Turbo Factor	7	3	7
Scan Time (min)	14:56	03:45	07:57

The following MRI datasets were analyzed:

- 30-day group (30D cohort): MRI were performed on 7 sheep (2 sites per sheep) at 22-28 days post-surgery, prior to sacrifice. Animals were sacrificed 28-30 days post-surgery for further analysis.
- 180-day group (180D cohort): MRI were performed on 11 sheep (2 sites per sheep) 169-175 days post-surgery. Animals were sacrificed between 178-183 days post-surgery for further analysis.
- 365-day group (365D cohort): MRI were performed on 10 sheep (2 sites per sheep) at 358-361 days post-surgery. Animals were sacrificed between 365-368 days post-surgery. Because micro-CT and histology are destructive, each animal was analysed at a single time point only; the three cohorts therefore comprised separate animals (30D: n = 7; 180D: n = 11; 365D: n = 10; Across the 28 animals, 56 defect sites were created. One control defect site from animal D180-03 was excluded due to insufficient MRI image quality, leaving 55 evaluable defect-site observations). No animal contributed to more than one time point.

Micro-CT analysis

Micro-CT imaging was performed using a Skyscan 1272 scanner (Bruker, Massachusetts, USA) with a voxel size of 10.8 μm, at room temperature. Samples were scanned and then placed back in 10% NBF. Projection images were processed with CT Pro 3D software to generate a floating-point 3D model. 2D models were also generated. Micro-CT imaging of the defect sites was performed on a Skyscan 1272 (Bruker) at a source voltage of 70 kV and a source current of 142 μA with a 0.5 mm aluminium filter; the defect sites of the acute animal (AC-01) were scanned on a Nikon XT H 225. Projection images were processed with CT Pro 3D software to generate 3D and 2D reconstructions. Trabecular bone volume (BV/TV, %) was calculated as the ratio of the volume of trabecular bone within a volume of interest (VOI) to the total VOI volume, with the VOI defined as the area of the original defect; all new bone within the VOI was included regardless of bone structure.

The following data were captured for each implanted site:

- **Trabecular bone volume (BV/TV, %)**: This parameter was calculated as the ratio between the volume of trabecular bone within the volume of interest (VOI) and the total volume of the VOI. The VOI was defined as the area corresponding to the original defect site. All new bone formed within the VOI, regardless of its structure, was included in the measurement of trabecular bone volume.
- **Defect area (mm^3)**: The volume of the defect site was measured in cubic millimeters (mm^3) to assess the extent of the original defect.

- **Amount of new bone (mm^3)**: The volume of new bone formed within the defect site was measured in mm^3. This measurement helped quantify the extent of bone healing and regeneration within the defect area.

The micro-CT imaging also allowed for the visualization and analysis of the bone structure and its integration with the surrounding tissue. Changes in the morphology of the bone defect and the formation of new bone were evaluated across different time points. These measurements provided a detailed assessment of the bone healing process in response to the implant.

For each time point, the imaging data enabled the analysis of the defect size, the amount of new bone formation, and the overall bone quality, thus offering a comprehensive view of the osteochondral repair process. The 3D models, along with 2D images, were evaluated to determine the progression of bone healing and the success of the implant in promoting bone regeneration.

Histological analyses

Histological evaluations were independently performed by Charles River Laboratories Montreal ULC (Quebec, Canada).

Once the trochleae were scanned, they were sectioned longitudinally into two approximate halves. This was done by sawing through the middle of the implant/defect site along the longitudinal axis, in the sagittal plane. Macroscopic images of the cut sections were captured. For further processing, one half of each trochlea was placed in formic acid for decalcification, and high-resolution Faxitron radiography was acquired to confirm decalcification, if needed. The decalcified samples were trimmed, processed, and infiltrated with paraffin. The other half of the trochlea was embedded in methyl methacrylate (MMA) for plastic embedding.

Using standard microtomy techniques, thin sections were cut, approximately 5 μm in thickness for the decalcified samples and between 6 and 10 μm for the non-decalcified samples. The target location for sectioning was the center of the defect site, whenever technically possible. The decalcified (paraffin-embedded) samples were stained with Hematoxylin and Eosin (H&E), Toluidine blue (TB), and Safranin-O Fast Green (SO), with three stained slides prepared for each sample. The non-decalcified (MMA-embedded) samples were stained with H&E and Toluidine blue, and two slides were prepared for each sample.

Histological images of the stained slides, including low- and high-magnification images for each defect/target site, were digitally captured and incorporated into the pathology report. The pathologist also noted any macroscopic observations surrounding the defect site, such as osteophytes or discoloration of the surrounding cartilage, which were grossly described if not already part of the tissue samples processed for histology. In addition, medial and lateral synovial tissues from the treated and non-treated stifle joints were processed similarly. These tissues were individually trimmed, paraffin-embedded, sectioned at approximately 5 μm, and stained with H&E for histological examination. All stained sections were examined by the Pathologist according to the protocols outlined in the Histopathology Section in the original Study [12]. Histological grading was performed by the Study Pathologist by light microscopy of the stained sections using the International Cartilage Repair Society (ICRS) II histological profiles (Mainil-Varlet et al.; Kon et al., modified); scores could range from 0% to 100% and were not limited to the defined table values. Toluidine blue (and Safranin-O, where present) slides were used for matrix staining, and H&E slides for all other parameters; bioresorption was graded using a semi-quantitative scheme. Sections were cut at ~5 μm (decalcified, paraffin) and 6–10 μm (non-decalcified, MMA), targeting the centre of the defect.

ICRS II Parameter

- **Subchondral bone reconstruction:**

For the evaluation of the femoral bone-cartilage defect sites, the International Cartilage Repair Society (ICRS) II histological scoring parameter was used, with a focus on subchondral bone abnormalities.

The following scoring was applied for the defect area in terms of subchondral bone reconstruction: 0% - No subchondral bone reconstruction; 33% - Minimal subchondral bone reconstruction; 67% - Moderate subchondral bone reconstruction; 100% - Normal appearance of subchondral bone. The scores could have ranged from 0% to 100% and were not limited to the defined scores mentioned above.

Additional histological information

Two additional parameters were also considered in this histological analysis:

- **New bone formation**:

This parameter quantifies the amount of newly formed bone tissue within the defect area, excluding the presence of bone marrow. It reflects the extent of mineralized tissue present.

- **Filling with new bone/marrow**:

This parameter assesses the extent to which the defect is filled by newly formed bone and marrow, including marrow tissue located between the trabecular bone, if present.

These two parameters were scored using the following scale:
0 = absent, 1 = minimal, 2 = mild, 3 = moderate, 4 = marked.

For the evaluation of each defect, two levels of slides were considered (decalcified vs. non-decalcified slides). The full evaluation was performed on each level, referred to as the medial half and lateral half. The results from both levels were averaged for the above-mentioned additional parameters. These scores were then combined to create a total score for each parameter at each test or empty site. The total scores for each parameter were used to summarize the data, including means and standard deviations. In cases where certain parameters could not be evaluated due to histological artifacts, those parameters were marked as ‘U’ (unavailable) in the data table. The total score for such parameters was derived from the second level of slides for that defect site, if available.

Statistical Analysis

All Likert, micro-CT, and histological variables were described by mean, standard deviation, median, minimum and maximum. The interrater reliability of the Likert rating was assessed through inter-item correlation matrices. To account for the effect of the group, partial Spearman's rank correlation coefficients between Likert questions, CNR, and other variables were calculated. Models were fitted based on significant correlations. The Shapiro-Wilk normality test was used to assess the normality of the examined variables. Spearman's rank correlation coefficients between Likert questions and other variables were calculated. Correlation strength was interpreted as follows: values between 0.00–0.19 were considered very weak, 0.20–0.39 weak, 0.40–0.59 moderate, 0.60–0.79 strong, and ≥0.80 very strong. The interrater reliability was assessed through inter-item correlation matrices, with Likert scale ratings used to quantify the agreement between the readers. For each correlation we reported a 95% confidence interval (CI) computed via the Fisher r-to-z transformation applied to the Pearson correlation of rank-transformed variables (standard approximation for Spearman). Specifically, after ranking both variables, the Pearson correlation r was Fisher-transformed. $z = 0.5 \cdot \ln[(1+r)/(1-r)]$, with standard error $SE\_z = 1/\sqrt{(n-3)}$; 95% CIs on z were $z \pm 1.96 \cdot SE\_z$ and then back-transformed to the correlation scale using $r = \tanh(z)$. Two-sided p-values are from Spearman tests with the corresponding sample (n) from each cohort.

To investigate the relationship between Likert questions (ordinal variables) and micro-CT and histological variables, we employed the Cumulative Link Mixed Models (CLMMs) with random intercept. Models were fitted using the function clmm from the R package ordinal version 2023.12-4.1 [24]. Since the sheep were sacrificed at different time points to obtain early results (30 days) and stabilized results (365 days), the range of all variables is inherently linked to the time point. Therefore, the effect of time was not included in the models, as it is already accounted for.

The fixed-effects structure was determined through likelihood ratio tests comparing different models. The micro-CT/histological variable and the intervention group were included as fixed effects, along with their interaction term. Additionally, random intercepts were specified for each animal. In the case of histological variables, high collinearity between fixed effects was discovered based on point-biserial correlation coefficient. Therefore, we settled for a simpler model containing only the histological variables and random effects. Tissue without the test article was set as the baseline in each model, and we report fixed effects that describe the effect of the micro-CT, or the histological variables controlled for the intervention group. The link function used was logit with an "equidistant" threshold. The proportional odds assumption was tested via the nominal_test() function from the ordinal R package. We present the fixed effects of models as Odds ratios (OR) with their lower and upper confidence intervals (CI) and corresponding p-values.

In the case of CNR analysis, linear mixed-effects models were fitted with a random intercept for animal and Kenward–Roger degrees-of-freedom correction using the function lmer from the R package lme4 version 1.1-35.1[25]. In all models, CNR parameter and group were

included as fixed effects, while we allowed individual intercepts for each animal. The interaction between CNR and the group was not included in the model because it was not significant and did not improve the model's fit. P–values lower than 0.05 were considered statistically significant.

Results (Extended):

MRI Likert Scale

The data from the study were analyzed for two primary Likert questions concerning bone repair tissue: (1) “Is the bone repair tissue signal intensity normal? – PD Signal Intensity Likert”, and (2) “Is the bone repair tissue mineralized? – T1 Bone Mineralization”. Table S2 presents the overall mean values, standard deviations, median, minimum and maximum for Likert questions and micro-CT and histological variables across time points (D30), (D180), and (D365).

Table S2. Summary statistics for Likert questions, micro-CT and histological variables across time points (D30), (D180), and (D365)

	Group	Timepoint	Mean	SD	Median	Minimum	Maximum
PD Signal Intensity	0	D30	2.71	0.49	3	2	3
	0	D180	3.91	1.87	3	2	7
	0	D365	5.70	1.89	6	3	9
	1	D30	3.00	0.58	3	2	4
	1	D180	8.70	0.48	9	8	9
	1	D365	8.80	1.32	9	6	10
	Overall		5.73	2.80	6	2	10
T1 Bone mineralization	0	D30	1.71	0.76	2	0	2
	0	D180	4.45	1.86	4	2	7
	0	D365	6.80	1.55	7	4	10
	1	D30	2.29	0.49	2	2	3
	1	D180	9.20	0.63	9	8	10
	1	D365	9.50	0.85	10	8	10
	Overall		6.04	3.20	7	0	10
Defect area (mm^3)	0	D30	229.06	22.28	226.75	196.70	266.05
	0	D180	228.60	14.89	235.76	202.49	244.41
	0	D365	223.89	12.30	224.73	204.71	240.91
	1	D30	233.53	16.23	224.82	219.07	262.85
	1	D180	230.46	13.56	230.50	208.72	253.03
	1	D365	225.52	6.54	225.12	215.77	239.27
	Overall		228.21	14.07	226.50	196.70	266.05
Amount of new bone (mm^3)	0	D30	20.17	10.99	16.98	7.87	34.86
	0	D180	84.94	19.94	79.15	60.92	122.65
	0	D365	93.59	25.40	84.74	64.75	142.87
	1	D30	65.31	19.10	60.88	45.62	94.73
	1	D180	113.33	19.00	108.63	91.50	158.96
	1	D365	116.44	17.75	114.97	93.08	143.00
	Overall		86.66	35.85	91.50	7.87	158.96
Trabecular bone volume (BV/TV, %)	0	D30	8.81	4.99	7.50	3.60	16.40
	0	D180	37.42	9.46	36.10	25.80	52.00
	0	D365	42.13	12.62	38.40	27.40	68.30
	1	D30	27.96	8.15	24.90	20.30	43.00
	1	D180	49.35	8.87	48.05	37.90	70.00
	1	D365	51.79	8.78	50.95	40.10	65.50
	Overall		38.21	16.28	40.10	3.60	70.00

	Group	Timepoint	Mean	SD	Median	Minimum	Maximum
Subchndral bone reconstruction (%)	0	D30	14.29	7.32	10.00	10.00	25.00
	0	D180	28.64	18.07	25.00	10.00	70.00
	0	D365	34.60	18.96	25.00	25.00	83.00
	1	D30	69.29	6.05	67.00	59.00	75.00
	1	D180	77.60	19.61	83.00	25.00	90.00
	1	D365	87.00	6.32	90.00	75.00	90.00
	Overall		52.58	30.65	59.00	10.00	90.00
New bone formation	0	D30	1.00	0.00	1.00	1.00	1.00
	0	D180	1.59	0.54	1.50	1.00	2.50
	0	D365	1.90	0.46	1.75	1.50	2.50
	1	D30	2.43	0.53	2.00	2.00	3.00
	1	D180	2.70	0.67	2.50	2.00	4.00
	1	D365	2.25	0.42	2.00	2.00	3.00
	Overall		2.00	0.72	2.00	1.00	4.00
Filling with new Bone/marrow	0	D30	1.07	0.19	1.00	1.00	1.50
	0	D180	1.73	0.79	1.50	1.00	3.50
	0	D365	1.80	0.82	1.50	1.00	3.50
	1	D30	3.43	0.61	3.50	2.50	4.00
	1	D180	3.75	0.42	4.00	3.00	4.00
	1	D365	4.00	0.00	4.00	4.00	4.00
	Overall		2.65	1.25	3.00	1.00	4.00
CNR PD	0	D30	8.85	2.61	9.66	3.82	12.09
	0	D180	1.44	1.23	1.05	0.48	4.18
	0	D365	4.22	2.81	2.77	1.31	8.30
	1	D30	5.39	2.28	5.89	2.01	7.69
	1	D180	0.23	0.16	0.21	0.00	0.49
	1	D365	1.49	0.71	1.50	0.53	2.69
	Overall		3.18	3.28	1.66	0.00	12.09
CNR T1	0	D30	7.86	2.21	8.10	3.21	9.88
	0	D180	1.70	2.09	0.99	0.03	7.48
	0	D365	4.42	2.72	3.54	1.44	10.23
	1	D30	8.32	3.09	8.84	3.64	12.49
	1	D180	3.86	2.95	2.62	1.32	9.35
	1	D365	5.40	2.85	4.51	2.51	10.80
	Overall		4.89	3.39	3.70	0.03	12.49

Interrater reliability

The interrater reliability analysis showed excellent agreement between the two readers regarding the Likert rating. As mentioned before, in cases where the Likert ratings differed by more than 2 points, a joint review was conducted to reach consensus; this occurred in three cases (D365_04_right, D30_01_right, and D30_05_right). The mean scores were similar for both readers, with Reader 1 having a mean of 5.88 (SD = 2.998) and Reader 2 having a mean of 5.86 (SD = 2.774). The inter-item correlation matrix revealed a very strong correlation of 0.949 between the two readers. The correlation coefficients within classes were significant, with a correlation of 0.947 for individual measures and 0.973 for average measures. Reader 1 showed excellent intra-reader agreement with an ICC of 0.963 (95% CI: 0.870–0.990, $p < 0.001$) and Spearman's $\rho = 0.973$ ($p < 0.001$). Reader 2 demonstrated good to excellent intra-reader agreement with an ICC of 0.920 (95% CI: 0.741–0.981, $p < 0.001$) and Spearman's $\rho = 0.918$ ($p < 0.001$).

Correlations:

Across all correlations, MRI Likert ratings showed clear associations with quantitative bone measures. For PD-weighted ratings, the correlation with micro-CT new bone volume was ρ=0.55 (95% CI, 0.33–0.71) and with BV/TV ρ=0.54 (0.32–0.70); PD ratings also correlated with histologic subchondral bone reconstruction, ρ=0.60 (0.40–0.75), and with filling by bone/marrow, ρ=0.46 (0.22–0.65), but not with histologic new bone formation alone, ρ=0.17 (−0.10–0.42). T1-weighted ratings demonstrated stronger associations with micro-CT (new bone volume ρ=0.70 [0.53–0.81]; BV/TV ρ=0.70 [0.53–0.81]) and with subchondral bone reconstruction on histology (ρ=0.68 [0.51–0.80]); correlations were moderate for filling by bone/marrow (ρ=0.55 [0.33–0.71]) and weaker but still significant for histologic new bone formation (ρ=0.29 [0.03–0.52]). Quantitative CNR analyses showed that PD-based CNR was inversely associated with micro-CT metrics (new bone volume ρ =−0.55 [−0.71 to −0.33]; BV/TV ρ =−0.53 [−0.70 to −0.31]) but not with histologic outcomes (CIs crossed zero). T1-based CNR correlations with both micro-CT and histology were small and non-significant (all 95% CIs included zero). Details are shown in Tables S3 and S4 and in Tables 1 and 2 (main paper).

Table S3. Correlation of PD-based Contrast-to-Noise Ratio (CNR) with Micro-CT and Histology

CNR PD	*ρ (95% CI)*	*Correlation strength*	*Significance (2-tailed)*	*N*
Amount of new bone (mm^3) - Micro-CT	−0.55 (−0.71 to −0.33)	Moderate	*< 0.001	55
Trabecular bone volume (BV/TV, %) - Micro-CT	−0.53 (−0.70 to −0.31)	Moderate	*< 0.001	55
Subchondral bone reconstruction - ICRS Histology	−0.20 (−0.44 to 0.07)	Weak	0.154	55
New bone formation - Histology	−0.12 (−0.37 to 0.15)	Very Weak	0.38	55
Filling with new bone/marrow - Histology	−0.24 (−0.48 to 0.03)	Weak	0.082	55

Table S4. Correlation of T1-based Contrast-to-Noise Ratio (CNR) with Micro-CT and Histology

CNR T1	*p (95% CI)*	*Correlation strength*	*Significance (2-tailed)*	*N*
Amount of new bone (mm^3) - Micro-CT	–0.17 (–0.42 to 0.10)	Very Weak	0.231	55
Trabecular bone volume (BV/TV, %) - Micro-CT	–0.16 (–0.41 to 0.11)	Very Weak	0.255	55
Subchondral bone reconstruction - ICRS Histology	–0.05 (–0.31 to 0.22)	Very Weak	0.708	55
New bone formation - Histology	0.18 (–0.09 to 0.43)	Very Weak	0.187	55
Filling with new bone/marrow - Histology	–0.06 (–0.32 to 0.21)	Very Weak	0.639	55

Cumulative Link Mixed Models (CLMMs) Likert – Micro-CT

The fixed effects showed a marginally significant association between "PD Signal Intensity" and "Amount of new bone" (OR = 1.026, 95% CI: 1.000–1.053, $p = 0.055$). Group (1) did not significantly contribute to the model (OR = 0.070, 95% CI: 0.002–2.254, $p = 0.133$). However, the interaction between "Amount of new bone" and Group (1) was significant (OR = 1.057, 95% CI: 1.016–1.099, $p = 0.006$).

Fixed effects demonstrated a significant association between "T1 bone mineralization" and "size of new bone" (OR = 1.045, 95% CI: 1.022–1.069, $p < 0.001$). Group (1) did not significantly contribute to the model (OR = 0.049, 95% CI: 0.002–1.354, $p = 0.075$). The interaction between "Amount of new bone" and Group (1) was significant (OR = 1.050, 95% CI: 1.011–1.090, $p = 0.012$).

Similarly, for the "Trabecular bone volume", the model showed a marginally significant association with "PD Signal Intensity" (OR = 1.056, 95% CI: 0.999–1.118, $p = 0.056$). Group (1) did not significantly contribute to the model (OR = 0.118, 95% CI: 0.004–3.202, $p = 0.204$). However, the interaction between "Trabecular bone volume" and Group (1) was significant (OR = 1.122, 95% CI: 1.031–1.221, $p = 0.008$).

Lastly, for the "Trabecular bone volume", the model showed a significant association with "T1 bone mineralization" (OR = 1.101, 95% CI: 1.047–1.158, $p < 0.001$). Group (1) did not significantly contribute to the model (OR = 0.082, 95% CI: 0.003–2.007, $p = 0.125$). The interaction between "Trabecular bone volume" and Group (1) was significant (OR = 1.106, 95% CI: 1.019–1.200, $p = 0.016$).

CLMM Likert – Histology

Due to high collinearity between histological parameters and the intervention group variables, the variable group was dropped in the final models. In model "T1 bone mineralization" – "Filling with new bone/marrow", the fixed effect variable was transformed by squared transformation to fulfill the parallel odds assumption. The model "PD Signal Intensity" – "New bone formation" was not fitted due to a nonsignificant relationship between the two variables.

All fitted models presented in Table S5 showed significant associations. Subchondral bone reconstruction significantly predicted PD Signal Intensity (OR = 1.066, 95% CI: 1.040–1.093, $p < 0.001$) and T1 bone mineralization (OR = 1.065, 95% CI: 1.040–1.091, $p < 0.001$). The PD Signal Intensity–New bone formation model was not fitted because the underlying bivariate relationship was not significant. New bone formation significantly predicted T1 bone mineralization (OR = 5.176, 95% CI: 2.145–12.490, $p < 0.001$). Filling with new bone/marrow significantly predicted PD Signal Intensity (OR = 5.098, 95% CI: 2.666–9.748, $p < 0.001$); for T1 bone mineralization, the squared filling term was significant (OR = 1.373, 95% CI: 1.214–1.554, $p < 0.001$).

Table S5. Random and fixed effects of cumulative link mixed models describing the relationship of the Likert score and Histology parameters.

PD Signal Intensity					T1 bone mineralization				
**Subchondral bone reconstruction - ICRS Histology**					**Subchondral bone reconstruction - ICRS Histology**				
Random effects					***Random effects***				
	Variance	SD				Variance	SD		
Animal	2.032	1.426			Animal	2.417	1.527		
Fixed effects					***Fixed effects***				
	OR	Lower CI	Upper CI	p-value		OR	Lower CI	Upper CI	p-value
Subchondral bone reconstruction	1.066	1.040	1.093	<0.001	Subchondral bone reconstruction	1.065	1.040	1.091	<0.001
**New bone formation - Histology**					**New bone formation - Histology**				
Random effects					***Random effects***				
	Variance	SD				Variance	SD		
Animal	-	-			Animal	1.630	1.277		
Fixed effects					***Fixed effects***				
	OR	Lower CI	Upper CI	p-value		OR	Lower CI	Upper CI	p-value
New bone formation	-	-	-	-	New bone formation	5.176	2.145	12.490	<0.001
**Filling with new bone/marrow - Histology**					**Filling with new bone/marrow – Histology**				
Random effects					***Random effects***				
	Variance	SD				Variance	SD		
Animal	3.269	1.808			Animal	3.755	1.938		
Fixed effects					***Fixed effects***				
	OR	Lower CI	Upper CI	p-value		OR	Lower CI	Upper CI	p-value
Filling with new bone/marrow	5.098	2.666	9.748	<0.001	Filling with new bone/marrow	1.373	1.214	1.554	<0.001

Linear mixed effects models CNR PD

For the outcome “Amount of new bone”, the fixed effects showed a significant negative relationship between the CNR PD calculation and new bone size (Estimate = -5.54, 95% CI: -7.91 to -3.16, p < 0.001), indicating that higher CNR PD values were associated with smaller new bone volumes. The group factor also had a significant positive effect (Estimate = 17.14, 95% CI: 7.76 to 26.52, p = 0.001), suggesting that belonging to the group (1) is associated with an increase of 17.14 mm3 in new bone amount, compared to the reference group.

For the outcome “Trabecular Bone volume BV/TV (%)”, the results showed a significant negative effect of CNR PD calculation on trabecular bone volume fraction (Estimate = -2.35, 95% CI: -3.47 to -1.24, $p < 0.001$). Additionally, the group variable had a positive influence (Estimate = 7.14, 95% CI: 2.78 to 11.49, $p = 0.002$), suggesting that group (1) is associated with higher trabecular bone volume of 7.14% compared to the group without the graft.

List of Tables - Electronic Supplementary Material

Table S1.

Parameters of the MRI Protocol.

Table S2.

Summary statistics for Likert questions and micro-CT and Histological variables across time points (D30), (D180), and (D365).

Table S3. Correlation of PD-based Contrast-to-Noise Ratio (CNR) with Micro-CT and Histology

Spearman correlations (ρ) between PD CNR ($|S_{new\ bone} – S_{surrounding\ bone}|/\sigma$) and bone quality outcomes. PD CNR shows significant inverse correlations with micro-CT new bone volume and BV/TV, while correlations with histologic measures are weak and non-significant. Two-sided p-values and 95% confidence intervals are reported (N=55).

Table S4. Correlation of T1-based Contrast-to-Noise Ratio (CNR) with Micro-CT and Histology

Spearman correlations (ρ) between T1 CNR and the same reference metrics. T1 CNR exhibits small, non-significant correlations with both micro-CT and histology, indicating limited discriminatory performance in this setting. Two-sided p-values and 95% confidence intervals are reported (N=55).

Table S5.

Random and fixed effects of cumulative link mixed models describing the relationship of the Likert score and Histology parameters.
